# The Histone Code of Senescence

**DOI:** 10.3390/cells9020466

**Published:** 2020-02-18

**Authors:** Harikrishnareddy Paluvai, Eros Di Giorgio, Claudio Brancolini

**Affiliations:** Department of Medicine, Università degli Studi di Udine. P.le Kolbe 4, 33100 Udine, Italy; hari.paluvai@student.unisi.it (H.P.); eros.digiorgio@uniud.it (E.D.G.)

**Keywords:** DNA damage, OIS, RS, SIPS, SAHF, SASP

## Abstract

Senescence is the end point of a complex cellular response that proceeds through a set of highly regulated steps. Initially, the permanent cell-cycle arrest that characterizes senescence is a pro-survival response to irreparable DNA damage. The maintenance of this prolonged condition requires the adaptation of the cells to an unfavorable, demanding and stressful microenvironment. This adaptation is orchestrated through a deep epigenetic resetting. A first wave of epigenetic changes builds a dam on irreparable DNA damage and sustains the pro-survival response and the cell-cycle arrest. Later on, a second wave of epigenetic modifications allows the genomic reorganization to sustain the transcription of pro-inflammatory genes. The balanced epigenetic dynamism of senescent cells influences physiological processes, such as differentiation, embryogenesis and aging, while its alteration leads to cancer, neurodegeneration and premature aging. Here we provide an overview of the most relevant histone modifications, which characterize senescence, aging and the activation of a prolonged DNA damage response.

## 1. Introduction

Aging is a physiological condition characterized by the functional deficit of tissues and organs due to the accumulation of senescent cells [1]. The key role of senescence in aging is well-established. Clearance of senescent cells in mouse models delays the appearance of age-related tissue and organ disfunctions [2,3]. Senescent cells are characterized by the permanent cell-cycle arrest sustained by the accumulation of cyclin-dependent kinase inhibitors/CDKi), like p16, p21 and p27, as well as by the release of cytokines, chemokines and soluble factors. This modified microenvironment is known as senescence-associated secretory phenotype (SASP) [4]. The senescence state is triggered by different stimuli/stressors. These include the shortening of the telomeres (replicative senescence), the oncogene-induced replication stress, the oncogene-induced senescence (OIS), the accumulation of misfolded protein and/or oxidative stress (stress-induced premature senescence, SIPS) [5].

The impairment of the non-homologous end joining (NHEJ) and homologous recombination (HR) repair mechanisms are common traits of senescent cells [6,7,8,9]. Moreover, a widespread epigenetic resetting characterizes senescent cells and sustains cell-cycle arrest and cellular survival, through the activation of (i) CDKi [10,11], (ii) tumor suppressors [12], and (iii) secretion of chemokines and cytokines, as well as the remodeling of the microenvironment [13].

Macroscopically, senescent cells are characterized by the formation of peculiar areas of heterochromatin, named as SAHF (senescence-associated heterochromatin foci), mainly at *E2F* loci [14]. However, SAHF do not characterize all senescent cells [15] and are not causally linked to the onset of senescence [16]. Other epigenetic features, like the distension of satellites (senescence-associated distension of satellites, SADS) [17], the re-activation of transposable elements, and of endogenous retroviruses (ERV) [18,19], seem to better qualify different types of senescence. Finally, aging appears to be marked by substantial re-arrangements of the nucleosomes, with the loss of histones H3 and H4 [20,21].

During senescence the epigenome undergoes temporal and sequential modifications that are mandatory to accomplish different cellular adaptations. Initially, this epigenetic resetting is mainly due to the accumulation of irreparable DNA damage. After this first wave of epigenetic modifications, the epigenome is remodeled and fixed in order to sustain the permanent cell-cycle arrest and to modulate the microenvironment.

## 2. The Epigenome of Replicative Senescence (RS)

The telomeric TTAGGG repeats at chromosome ends protect the genome from degradation and distinguish natural chromosomes ends from double-strand breaks (DSBs) [5,22,23]. Histone and non-histone (Shelterin) proteins sustain the folding of telomeric repeats in high-order chromatin structures that acquire a G-quadruplex shape as a consequence of Hoogsteen base pairing between consecutive guanines [24]. The loss of active telomerase complexes in somatic human cells blocks the lengthening of the telomeric ends. As a consequence, for each successful cell division, telomeres get shorter and cell proliferation is restricted. This phenomenon is defined as replicative senescence (RS) [25]. The accumulation of irreparable DNA damage triggered during RS leads to permanent cell-cycle arrest and is considered among the main driving forces of aging [22].

### 2.1. Histone Variants

The progressive accumulation of double-strand breaks (DSBs) at the chromosome ends is coupled with a deep epigenetic resetting that can be observed in pre-senescent cells, even distal from telomeres. This epigenetic repertoire builds up an epigenetic clock that dictates the replicative potential of human cells [26]. Late passage IMR90 and WI38 human fibroblasts are characterized by a reduced expression of core histone H3 and H4 [21], of the linker histone H1 [27] and of the histone chaperons ASF1A/B and CAF1-p150/p60 [28]. While the decreased levels of H3 and H4 are due to reduced neosynthesis and increased mRNA degradation [21,29], H1 is post-translationally regulated [27]. Moreover, alternative spliced histone mRNAs belonging to the HIST1 cluster are reported to be accumulated in quiescent and RS-arrested human fibroblasts [30].

The epigenome of RS cells is also characterized by the deposition, at certain genomic loci, of the histone variants H3.3 [31], H2A.J [32] and by the release of genomic DNA from H2A.Z [33,34,35] (Table 1). This redistribution results in chromatin remodeling and promotes the transcription of (i) tumor suppressors [30,31], (ii) inflammatory genes marking the SASP, [32] and iii) the cleavage of H3.3, which mediates the repression of E2F/RB target genes [31]. While in senescence, the HIRA-mediated deposition of H3.3 sustains cell-cycle arrest [31], and in embryonic stem cells ATRX and DAXX recruit H3.3 to repress the transcription of endogenous retroviruses (ERVs) [36].

It is possible that the redistribution of H3.3 between the proliferating and senescent cells, which depends on the detachment from ATRX/DAXX and the complexing to HIRA, is at the basis of the activation of ERVs observed in senescence [37] and aging [38].

The regulated deposition of all these histone variants is necessary [30] and sufficient to sustain cell-cycle arrest [31]. Interestingly, genes encoding these histone variants are frequently mutated in cancer, in confirmation of their tumor-suppressive properties [30,39].

### 2.2. SAHF an Open Question?

The HIRA chaperone complex (HIRA/UBN1/CABIN1) controls both the deposition of H3.3 [30] and SAHF formation [40]. SAHF accumulation of H3K9me3/H3K27me3/macroH2A blocks on E2F loci characterizes OIS and cells undergoing oncogene-induced replication stress [15]. However, RS is not uniformly characterized by the formation of SAHF [41]. In fact, focused heterochromatinization of *E2F* loci maintains cell-cycle arrest, also in cells described as SAHF-negative (e.g., BJ and MEFs) [41,42]. SAHF are defined as DAPI-dense nuclear regions characterized by the presence of a central core of condensed chromatin, enriched for H3K9me3 and macroH2A. This core is surrounded by a peripheral ring of H3K27me3 [43,44]. SAHF formation requires p16/INK4 and consists of a deep and focused heterochromatin re-organization [45]. This reorganization is HMGA1/ASF1/HIRA-dependent [40,46] and is triggered by the GSK3β-mediated HIRA re-localization at PML bodies [47]. Even though SAHF dismantling, achieved through HMGA1 [46], ASF1 [40] or GSK3β knockdown [48], allows senescence escape, BJ fibroblasts and Hutchinson–Gilford progeria syndrome (HGPS) cells enter senescence with minimal or no signs of SAHF formation. On the opposite, the SAHF formation in HMEC and MCF10A mammary cells in response to H-RAS/G12V over-expression fails to bring the cells to senescence [15]. Whether SAHF formation is only due to the arising of replication stress and could act as a barrier to DNA double-strand breaks spreading [15], or could mark chromatin regions stitched between remodeled LAD domains [45], needs further investigation. It is possible that a better definition of the SAHF, achieved through the improvement of the resolution of confocal nanoscopy and of ChIP-seq histone mapping, will clarify the debated role played by SAHF in senescence.

### 2.3. Histone Modifications

A global decrease in H3K9me2/3 and H4K20me but increase in H3K9me1 levels in gene bodies characterize RS in human fibroblasts [49,50]. By contrast, the heterochromatin marker H3K27me3 and the euchromatin marker H3K4me3 are mostly redistributed with respect to proliferating cells (Table 2).

This redistribution correlates well with the expression profile of senescent cells [51,52,53]. A different behavior was reported for the repressive mark H4K20me3 and the activating mark H4K16ac. Although they are enriched in the regulative elements of genes modulated during RS, they do not correlate with gene expression changes observed in senescent cells [30,54]. This apparent paradox could stem from the masking effect imposed by the senescence-specific activation of super-enhancers (marked by H3K27ac/H3K4me1) and the activation of neighbor genes involved in SASP and metabolism [55].

A detailed comparison of H3K4me3 and H3K27me3 levels in proliferating and senescent human fibroblasts evidenced large-scale chromatin modifications during RS [56]. In senescent cells the augmented levels of H3K4me3 and H3K27me3 frequently co-localize in areas defined “mesas” that extend for hundreds of kilobases. Larger domains of RS genome (up to 10Mb) and defined “canyons” are characterized by decreased levels of H3K27me3 [56]. H3K4me3 and H3K27me3 mesas colocalize in LMNB1-associated domains and overlap DNA hypomethylation. Instead, canyons are enriched in gene bodies and enhancers and H3K27me3 loss correlates with the up-regulation of senescent transcriptional programs [56].

### 2.4. Nuclear Lamins

The loss of LMNB1 typifies all senescence conditions [57] and triggers a deep re-modelling of lamina-associated domains (LADs) [44,56]. LADs re-modelling contributes to re-organizing not only heterochromatin and SAHF [44], but also euchromatin (eLADs) [58]. Moreover, the knockdown of *LMNB1* in proliferating cells promotes the premature senescence and gives rise to a H3K4me3/H3K27me3 re-organization similarly to what observed in RS, OIS and HGPS [56]. However, LMNB1 re-expression in RIS senescent cells does not overcome the proliferation of arrest and does not repair nuclear membrane blebbing [59]. It is still unknown if the re-expression of LMNB1 in senescent cells is strong enough to re-establish normal LAD domains or if the co-expression of an epigenetic regulator is needed. Similarly, the ectopic expression of lamina-associated polypeptide 2α restores the proliferation of HGPS cells [60], characterized by the expression of the progerin form of LMNA [61,62]. Unfortunately, the impact of LAP2α expression on LAD domains has not been explored yet. The re-localized LAD domains in RS are characterized also by a general DNA hypomethylation that affects *LINE* and *SINE* repetitive elements and pericentromeric satellites [17]. This hypomethylation triggers the general distension of the chromatin associated to these genomic regions (senescence-associated distension of satellites or SADS) and their de-repression [17]. The activation of centromeric and pericentromeric satellite repeats is associated with their exclusion from nucleoli compartments, while all the other nucleoli-associated domains (NADs) are significantly altered in senescent cells [63].

Despite LADs redistribution, 3D chromatin organization of human dermal fibroblasts (HDFs) is only partially altered when proliferating, quiescent and senescent cells are compared. A modest gain of short-range and the loss of long-range intra-chromosome interactions in permanently arrested cells was observed [64]. More evident in quiescent and senescent cells is the switch of topologically associated domains (TADs) from euchromatin areas (Hi-C compartment A) to heterochromatin (Hi-C compartment B) and vice versa. This remodeling reflects the transcriptional status of cell-cycle-associated genes [64]. Most of the heterochromatinization is due to condensin mobilization [65] and PRC2 (EZH2) deposition [66], while MLL1 plays a role in mediating euchromatinization [67,68]. Moreover, RS cells lose the TADs associated to the telomeres [69].

### 2.5. The CpG Methylation Clock

In general, DNA methylation during aging progressively involve both hypomethylation and hypermethylation events. Importantly, the methylation status of a limited number of well-defined CpG islands associate well with human aging [26]. The quantification of the methylation rate of these CpG island allows a good prediction of human biological age [26,70,71,72]. According to these estimations, the methylome of men ages 4% faster than women [70]. Moreover, the methylation clock is accelerated in patients affected by neurodegenerative diseases [73,74,75], chronic stress and insomnia [76], as well as in two premature aging disorders like Werner’s syndromes and Hutchinson–Gilford Progeria syndrome [76,77], while it is subverted in cancer [78,79].

Aging is characterized by enhancer hypomethylation [41] and this correlates with the loss-of-function of stem cells [80]. Similar changes in the methylation prolife also characterize RS [81], and global hypomethylation characterizes both genomic and mitochondrial DNA [82]. CpG hypermethylated regions are associated with H3K27me3, H3K4me3 and H3K4me1, whereas hypomethylation is observed in the constitutive heterochromatin and lamina-associated domains (LADs) [83].

## 3. The Epigenome of Oncogene-Induced Senescence (OIS)

The expression of a certain number of oncogenes (K-RAS and H-RAS, BRAFV600E, myrAKT1, STAT5, nuclear HDAC4, N1ICD, ERBB2 and β-catenin) [84,85,86,87,88,89,90,91,92] or ablation of tumor suppressors (PTEN, APC and AXIN) in normal cells triggers a permanent and premature cell-cycle arrest defined as OIS [93,94]. OIS can occur also in the presence of ectopic TERT expression [6]. The most studied model of OIS is the RAS-induced senescence (RIS), obtained by over-expressing oncogenic mutants of RAS in fibroblasts, melanocytes and retinal pigment epithelial cells. The consistency of the RIS model has been validated in mice, after conditional induction of the monoallelic expression of K-RAS/G12V. Mice develop lung adenomas characterized by the accumulation of senescent cells [95]. Similarly, premature senescence blocks the spreading of oncogenic lesions in BRAF/V600E expressing melanocytic nevi [89]. Curiously, RAS fails to induce senescence in immortalized human mammary epithelial cells (HMECs), even after the ectopically expression of p16/INKa [96,97]. This failure has been associated to defects in the TGFβ signaling pathway [97].

It is generally accepted that the premature cell-cycle arrest characterizing OIS is elicited by the accumulation of irreparable DNA damage. In fact, the abrogation of ATM signaling allows senescence escape [6]. It is important to note that the DDR dependence was observed for RAS but its involvement in the case of other oncogenes, such as NOTCH and AKT1, needs further validations [90,98]. As explained above, RIS is characterized by the accumulation of SAHF [14]. However, the oncogenic activation of *AKT1* and *NOTCH* or the knockdown of *PTEN* trigger OIS without SAHF formation [85,86,98]. In the case of *NOTCH*, this is due to the repression of HMGA1 [90]. Differently, in the case of *AKT1* it was hypothesized a mechanism operating through GSK3β inhibition. In fact GSK3β controls the phosphorylation-dependent HIRA sub-compartmentalization [99]. In this respect, a similar defect in HIRA signaling prevents SAHF formation in senescent murine embryonic and skin fibroblasts expressing RAS, but not to the accumulation of nuclear macroH2A.1 [42]. The deposition of the histone variant macroH2A.1 is not only required for SAHF formation. It also sustains a chromatin re-organization that allows the focused histone acetylation and the expression of the typical SASP cytokines and chemokines [100]. Moreover, the knock-down of macroH2A.1 in H-RAS/V12 IMR90-expressing cells not only blunts SASP, but also decreases the phosphorylation of γH2AX [100].

A common property of OIS cells is the loss of LMNB1 [57]. This causes a deep re-organization of LAD domains [44], similarly to what was observed during RS [56]. As a consequence, OIS is characterized by the re-localization and re-organization of LAD-associated heterochromatin domains [43,101]. This re-organization is sculptured in the distribution of H3K4me3 and H3K27me3 “mesas” and H3K27me3 “canyons”, as described above for RS cells [56]. Interestingly, the expansion of H3K27me3 “canyons” achieved though EZH2 repression sustains OIS by promoting the expression of cytokines [56] and of CDKi [66,102,103].

Similarly to RS, the histone variant H3.3 [31,104] and its Cathepsin L-mediated cleavage product H3.3cs1 [104] are deposited in RIS cells in the regulative elements of RB/E2F target allowing the permanent removal of H3K4me3 [31] and the increase in H3K9me3 levels [14]. Contemporary, the removal of H2A.Z and the de-methylation of H3K27me3 at tumor suppressor loci sustains cell-cycle arrest [33,66]. The inflammatory response is achieved through the deposition of the histone variant H2A.J [32] and the binding of HMGB2, which excludes these loci from SAHF [41]. HMGB2 also allows the H3K4 trimethylation mediated by the methyltransferase MLL1 [53]. Moreover, SASP loci are localized in newly formed super-enhancers that require the binding of BRD4 to promote their transcription [105,106].

Finally, OIS in human fibroblasts is characterized by the spatial rearrangement of pre-existing heterochromatin that give rise to SAHF [45]. Differently, HGPS cells are refractory to SAHF formation [45,107,108] and to heterochromatin focusing [45], probably because the huge alterations in the lamina compartment of these cells prevents the proper heterochromatin 3D-structure organization [109]. A similar displacement of H3K9me3 from LADs is observed in OIS, but it is followed by an increase in local interactions between H3K9me3 domains and sharp heterochromatinization [45]. Curiously and differently from RS and aging, OIS cells do not display any changes in CpG island methylation levels [110].

### Different Types of OIS?

While studies are increasingly describing RIS epigenetics, detailed data about the epigenetic modifications in other types of OIS are unavailable. Additional data are desirable since increasing evidences highlight the key roles played by epigenetic regulators in maintaining OIS and counteracting oncogenic transformation [84,111,112,113,114,115,116]. For example, in melanomas the activation of H3K9me3 demethylases (LSD1 and JMJD2C) selectively de-represses E2F target genes, allowing senescence escape and tumorigenesis [117]. This result reinforces the idea that a better investigation of the epigenetic mechanism that sustains the early step of tumorigenesis is desperately needed.

## 4. The Epigenome of Stress Induced Premature Senescence (SIPS)

SIPS is characterized by the accumulation of ROS (reactive oxygen species), due to mitochondrial disfunctions, ER stress or the exogenous administration of oxidative compounds [118,119,120]. Even though SIPS onset is independent from telomere attrition, ROS accumulation can cause telomere disfunction and fusion in primary cells, thus sustaining cell-cycle arrest [120,121]. Accordingly, murine embryonic fibroblasts (MEFs) cultured in normoxia undergo premature senescence due to the accumulation of ROS-induced DNA damage, while the same cells cultured in hypoxia tend to indefinitely proliferate in virtue of the long telomeres [122].

When human cells are exposed to oxidative stress in vitro, they undergo stochastic transcriptional changes which resemble aged tissue. Recently, it has been demonstrated that oxidative stress contributes not only to aging but also to age-related diseases [123]. Moreover, the accumulation of 8-oxo-7,8-dihydro-2′-deoxyguanosine (8-oxodG) has been observed in the liver of aged mice [124]. Global histone methylations of H3K4, K27 and K9 are increased when BEAS-2B cells are exposed to H_2_O_2_, and preincubation with ascorbate reverse these changes [125]. These evidences are confirmed also in other contexts [126]. The general heterochromatinization observed after H_2_O_2_ treatment is achieved in two steps. Firstly, by reducing acetylation (H3K9ac, H4K8ac, H4K16ac) [125,127] in a HDAC-dependent manner [127,128]. Secondly, by recruiting histone methyltransferases (HMTs) and inducing H3K27me3, H3K9me3 and H4K20me3 [127,129]. Chromatin condensation is an attempt to preserve the DNA from genotoxicity [130].

The downstream pathways that lead to cell-cycle arrest in cells exposed to oxidative stress imply the up-regulation of CDKi, the DDR response and SASP production, similarly to cells undergoing RS [131,132]. In addition, mitochondrial dysfunctions in IMR90 human fibroblasts lead to the ROS–JNK retrograde signaling pathways, which promote SASP and drive cytoplasmic chromatin fragments (CCFs) [133]. Importantly, the epigenetic homeostasis perturbation, achieved through HMTs or HDACs inhibition [129], is reported to expose cells to endogenous ROS production [134] or to trigger and sustain the senescence induced by the treatment with oxidative compounds [135]. In particular, some ROS generators inhibit PRC2 methyltransferases to allow the focused demethylation and transcriptional activation of CDKi [132]. Similarly, the senescence entry of *PAK2* knocked-down MEFs cultured in normoxia is delayed because of the decreased deposition of H3.3 on CDKi loci [136]. At the DNA level, SIPS is characterized by a global DNA hypomethylation that only partially overlaps with the one observed during RS [81].

The altered expression and activation of epigenetic regulators allows cancer cells to escape cell-cycle arrest and to proliferate even in the presence of high levels of oxidative and replication stress, perpetuating the accumulation of DNA damage from generation to generation [137]. In short, histone variants and histone posttranslational modifications taking place during senescence are listed in Table 1 and Table 2.

**Table 1:** Histone variants that characterize senescence. RS: Replicative senescence; OIS: Oncogene induced senescence; SIPS: Stress induced premature senescence; SASP: Senescence associated secretory phenotype; ↑: Increased expression; ↓: Decreased expression; →: No change; NI: Not investigated.

Histones and Histone Variants	Role during Senescence	Changes during RS	Changes during OIS	Changes during SIPS	References
**H1**	Chromatin condensation	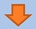	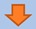	NI	[27]
**H2A**	Chromatin condensation	Increased ratio H2A2/H2A1	NI	NI	[15,40]
**H3, H4**	Chromatin condensation	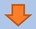	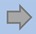	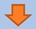	[21]
**H2A.X**	Sensor of DNA damage	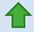	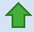	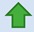	[21]
**H2A.J**	Promote SASP	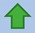	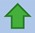	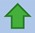	[32]
**H2A.Z**	Regulation of CDKi	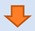	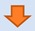	NI	[33,34]
**H3.3, H3.3cs1**	Gene activation, silencing, chromosome segregation	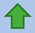	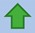	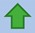	[31,36,104]
**H3.1**	Gene activation, silencing, chromosome segregation	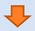	NI	NI	[21]

**Table 2:** Histone post-translation modifications (PMTs) observed in senescence. RS: Replicative senescence; OIS: oncogene induced senescence; SIPS: Stress induced premature senescence; SASP: Senescence associated secretory phenotype; ↑: Increased expression; ↓: Decreased expression; →: No change; NI: Not investigated.

Histone PMTs	Enzymes Involved	Role during Senescence	Changes during RS	Changes during OIS	Changes during SIPS	References
**H3K9me3**	KDM4A KDM4B	Heterochromatin	↓Relocalized in focused area	↑Relocalized in focused area	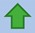	[43,45,128,129]
**H3K27me3**	KDM6B (JMJD3)	Heterochromatin	↓Relocalized in focused area	↓Relocalized in focused area	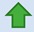	[43,44,45,102,103,127,128,129]
**H4K20me3**	Increased activity of Suv420h2	Heterochromatin gene repression	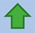	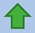	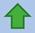	[54,127]
**H3K4me3**	KMT2/KDM5D/KDM2B	Euchromatin, gene activation	Relocalized	Relocalized	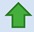	[56,125]
**H4K16ac**	(MOF) Histone acetyltransferase	DNA repair, chromatin compaction	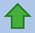	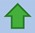	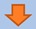	[125,127]
**H3K9ac**	HAT/p300	Euchromatin,	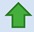	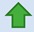	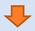	[55,125,127]
**H4K8ac**	HAT/p300	Euchromatin	NI	NI	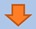	[125,127]

## 5. The Epigenome at DSBs and during DDR: Early Epigenetic Events in the Senescent Response

Double-strand DNA breaks cause massive epigenetic changes. Immediately, at the damaged sites, the DNA unwraps from the histones and the chromatin undergoes a de-structuration, thus losing compactness [138]. This response is achieved mainly through the chaperone-mediated nucleosomes disassembly [139]. Functionally, it allows the recruitment of proteins involved in DDR. Structurally it causes significant topological alterations in the DNA fiber. These modifications are limited by the subsequent heterochromatinization upstream and downstream from the damage site, which restrains relaxation [140].

The phosphorylation by PIKK kinases (ATM, ATR and DNA-PK) of ser 139 of H2AX (γH2AX) is the widespread histone modification that extends for megabases around a DSBs. γH2AX acts as a platform for the recruitment of MDC1 and 53BP1. This recruitment is sustained by the accumulation at the damaged site of DDR RNAs [141]. The general relaxation of the chromatin is achieved through different mechanisms, which include (i) the RNF8/Ubc13/HUWE1 ubiquitylation of Histone H1 [142,143], (ii) the RNF168 mediated K63-ubiquitylation of H2A/B and H2AX [144], and (iii) the PARylation-dependent, proteasomal degradation of H1.2 [145].

In addition to these huge chromatin remodeling, local histone modifications surrounding the DSBs take place in cells undergoing NHEJ or HR, thus sculpturing an epigenetic pattern specific for each of the two repair mechanisms [138]. The activation of NHEJ is supported by a general chromatin expansion around the DSBs, by the deposition of the histone variants H3.3 [146] and H2AZ [147] and by the monoubiquitylation of H2BK120. This monoubiquitylation promotes the appearance of H3K4me3 and the binding of the SWI/SNF remodeling complex. Moreover, the TIE2-mediated phosphorylation of H4Y51 [148], as well as the RNF20/40-mediated H2BK120ub [149] and the RNF168-mediated H2AK15ub [144] act as platforms to recruit DDR proteins, like ABL1 [148], Ku70/80 [149] and 53BP1 [150,151]. The latter modification and the successful recruitment of 53BP1, which controls end re-sectioning, dictate the choice of the NHEJ pathway in spite of HR [152,153]. H4K20me2 is another anti-resection modification that reinforce 53BP1 binding to the chromatin [154]. However, the co-presence of H2AK15Ub and H4K20me2 in the proximity of the DSBs buffers NHEJ processing by recruiting the HAT Tip60/KAT5, which triggers H2AK15ac and 53BP1 displacement. An activity that favors the establishing of HR [155]. 53BP1 displacement seems to occur during S/G2 transition, as in G1 cells 53BP1 stably occupying the HR sites [156]. The general loss of nucleosome occupancy achieved in proximity to DSBs is counterbalanced by the distal accumulation of H3K36me3 [156]. This PTM can be used as a scaffold for the binding of HDACs, thus ensuring the transcriptional silencing of genomic loci affected by DSBs [157,158].

A different epigenetic environment is associated to the activation of the HR pathway [158]. In this context, macroH2A is found to be more abundant than H2AZ, the acetylation of H2BK120 overcomes ubiquitylation in a SAGA complex-dependent manner [159] and γH2AX is more spread [156]. As explained above, Tip60/KAT5 acetylates H2AK15, thus displacing 53BP1 [155]. Tip60/KAT5 also maintains the acetylation of H4K16, which is required for keeping an open chromatin status [160] at the damaged site. An opposite epigenetic mark, H3K9me2, is required for the BARD1/HP1γ mediated retention of BRCA1 [161] and to allow the loading of the pro-resection factor CtIP [162]. In active genes affected by DSBs, the loss in H3K79me2 and of H4 acetylation levels [156], as well as the recruitment of macroH2A [163] and of repressive protein complexes (HP1,KAP1,SUV39H1,PRDM2,HDACs), seems to compensate for the general loss of histones that characterizes the damaged sites [164]. The spatio-temporal regulation of chromatin remodeling at DSBs is achieved and sustained also by the deposition of other histone variants, like the HIRA-assisted H3.3 and the CAF-1-mediated H3.3 [23].

In summary, from the literature emerges a bleeding/vasoconstriction model in which the chromatin expands and becomes flexible at the damaged sites to accommodate the repair complexes, but heterochromatinization and the creation of a transcriptional repressive environment is required later on to allow the repair before restarting the transcription. The successful prediction of DSBs by looking at the histone positioning and PTMs [165], the emerging roles played by epigenetic regulators in DDR as well as the impact of epigenetic drugs on DDR [166] confirm that the epigenetic status of the chromatin not only identifies sites of genome fragility, but it is also causally linked to the successful repair [23].

## 6. An Anti-Apoptotic Response Sustains the Survival of Damaged Cells Exposed to Irreparable DNA Damage

Senescent cells display an increased resistance to pro-apoptotic stimuli that is achieved through the up-regulation of the pro-survival gene *Bcl2* and *Bcl-XL* and the down-regulation of the pro-apoptotic genes *Bid* and *Bax* [127]. The TP53-dependent up-regulation of the CDKi p21 plays a key role in sustaining the survival of damaged cells [167], while DNMT3a and HDAC1 are recruited on the p21 promoter to switch off its expression during apoptosis [168]. The increase in p21 promotes the cell-cycle arrest allowing the activation of DDR; cellular proliferation is subsequently permanently locked through the activation of INK4 CDKi (mainly p16 and p15) [169]. Many of the anti-apoptotic functions of p21 are achieved through the modulation of TFs (p300, STAT, JUN and TP53) [170] and are sustained by an epigenetic reprogramming [127]. In senescent cells, high levels of H4K20me3 and low levels of H4K16ac keep down the transcription of pro-apoptotic genes, while the increased acetylation of H4K16 characterizes pro-survival loci [127]. The heterochromatinization that surrounds and borders DSBs enhances these pro-survival responses and any relaxation of these structures, obtained for example through HDAC inhibition, triggers apoptosis [6]. Finally, the mTORC1 and PGC-1β dependent metabolic reprogramming [171] observed in senescent cells and in different models of aging [5] ensures energy supply.

Since the epigenetic regulators require cofactors that are produced in a large majority in mitochondria [172,173], any mitochondrial dysfunction can affect both the transcriptional and the metabolic fitness of the cells and lead to senescence even in absence of DNA damage (MiDAS, mitochondrial dysfunction-associated senescence) [171].

Severe and acute stresses induce cell death, while prolonged and mild insults lead to cellular senescence and survival, probably because the cells have the time and a not completely compromised ability to mount the epigenetic, transcriptional and metabolic responses that characterize them [174].

## 7. Final Considerations: The Link between Senescence, Aging, Epigenetics and DDR

The accumulation of DSBs is a general hallmark of senescence and aging [6]. The main endogenous sources of DSBs are telomere attrition and replicative stress. Replication stress is commonly observed in RS, OIS and aging. In all these conditions cells slow down DNA synthesis and replication fork progression. However, the reduced replication fork speed activates dormant origin to preserve replication timing during replication stress [175]. This adaptive response allows the maintenance of an unaltered replication timing also in cells entering senescence [176]. On the other side it exposes common fragile sites (CFSs), which are genomic loci more prone to breakage after DNA polymerase inhibition, and the accumulation of genomic alterations. CFS alterations are typically observed in pre-neoplastic lesions [177]. Similarly, cells exposed to genotoxic agents (e.g., chemotherapeutic agents, pollutants and toxins) or to oxidative stress activate the DDR that frequently leads to cell-cycle arrest.

Whatever the origin, the accumulation of irreparable DNA damage gives rise to a univocal response characterized by the activation of tumor suppressors and CDKi and by the release of pro-inflammatory cytokines [5].

Global histone loss, as well as the focused deposition of histone variants (H2AX, H2AZ, H2AJ, H3.3 and macroH2A) and the redistribution of H3K4me3, H3K27me3 and H3K36me3 characterize both DDR, DSBs and senescence. The chromatin remodeling observed in different senescence models seems to represent a temporal and spatial evolution of what is observed after a short-time treatment of the cells with DNA damaging agents. Although it is clear that an altered epigenome can expose cells to DSBs [134] and that epigenetic regulators control the fate of damaged cells [15,55,66,115,178,179,180], investigations on the epigenetic inheritance in daughter cells coming from DNA-damaged cells are still in their infancy [177].

Cancer cells appear as forgetful cells that have lost the epigenetic memory of a healthy genome. Aging seems to be predisposed to this memory loss. One of the major challenges of the future regarding the treatment of aging and cancer, will be the identification of the framework of epigenetic changes that can restore this memory.
